# Rapid eradication of colon carcinoma by *Clostridium perfringens* Enterotoxin suicidal gene therapy

**DOI:** 10.1186/s12885-017-3123-x

**Published:** 2017-02-13

**Authors:** Jessica Pahle, Lutz Menzel, Nicole Niesler, Dennis Kobelt, Jutta Aumann, Maria Rivera, Wolfgang Walther

**Affiliations:** 1 Experimental and Clinical Research Center, Charité University Medicine, Lindenberger Weg 80, 13125 Berlin, Germany; 2 Max-Delbrück-Center for Molecular Medicine, Rober-Rössle-Str.10, 13125 Berlin, Germany; 3 Experimental Pharmacology & Oncology (EPO) GmbH Berlin, Rober-Rössle-Str. 10, 13125 Berlin, Germany

**Keywords:** *Clostridium perfringens* enterotoxin (CPE), Colon cancer, Gene therapy, Suicide gene

## Abstract

**Background:**

Bacterial toxins have evolved to an effective therapeutic option for cancer therapy. The *Clostridium perfringens* enterotoxin (CPE) is a pore-forming toxin with selective cytotoxicity. The transmembrane tight junction proteins claudin-3 and -4 are known high affinity CPE receptors. Their expression is highly upregulated in human cancers, including breast, ovarian and colon carcinoma. CPE binding to claudins triggers membrane pore complex formation, which leads to rapid cell death. Previous studies demonstrated the anti-tumoral effect of treatment with recombinant CPE-protein. Our approach aimed at evaluation of a selective and targeted cancer gene therapy of claudin-3- and/or claudin-4- expressing colon carcinoma in vitro and in vivo by using translation optimized CPE expressing vector.

**Methods:**

In this study the recombinant CPE and a translation optimized CPE expressing vector (optCPE) was used for targeted gene therapy of claudin-3 and/or -4 overexpressing colon cancer cell lines. All experiments were performed in the human SW480, SW620, HCT116, CaCo-2 and HT-29 colon cancer and the isogenic Sk-Mel5 and Sk-Mel5 Cldn-3-YFP melanoma cell lines. Claudin expression analysis was done at protein and mRNA level, which was confirmed by immunohistochemistry. The CPE induced cytotoxicity was analyzed by the MTT cytotoxicity assay. In addition patient derived colon carcinoma xenografts (PDX) were characterized and used for the intratumoral in vivo gene transfer of the optCPE expressing vector in PDX bearing nude mice.

**Results:**

Claudin-3 and -4 overexpressing colon carcinoma lines showed high sensitivity towards both recCPE application and optCPE gene transfer. The positive correlation between CPE cytotoxicity and level of claudin expression was demonstrated. Transfection of optCPE led to targeted, rapid cytotoxic effects such as membrane disruption and necrosis in claudin overexpressing cells. The intratumoral optCPE in vivo gene transfer led to tumor growth inhibition in colon carcinoma PDX bearing mice in association with massive necrosis due to the intratumoral optCPE expression.

**Conclusions:**

This novel approach demonstrates that optCPE gene transfer represents a promising and efficient therapeutic option for a targeted suicide gene therapy of claudin-3 and/or claudin-4 overexpressing colon carcinomas, leading to rapid and effective tumor cell killing in vitro and in vivo.

**Electronic supplementary material:**

The online version of this article (doi:10.1186/s12885-017-3123-x) contains supplementary material, which is available to authorized users.

## Background

The incidence of colorectal cancer is increasing and is associated with the fourth highest cancer associated mortality rate worldwide [1, 2]. Despite advances in chemotherapy, radiotherapy and the development of new drugs, the prognosis for patients remains poor. Therefore, new targets and therapeutic substances are desperately needed [3, 4].

One promising strategy may be targeted suicidal cancer gene therapy, including an approach by which foreign toxic molecules are specifically delivered to tumor cells [5, 6]. Attractive candidates include bacterial toxins, which have demonstrated efficient cell-killing capacity in several in vitro and in vivo studies [7–10]. Pore-forming bacterial toxins, such as streptolysin O (*Streptococcus pyrogenes*) and *Clostridium perfringes* enterotoxin (CPE), are of particular interest [11–14].

Strain A *Clostridium perfringens,* an anaerobic gram-positive bacterium, produces the CPE protein, associated mainly with food poisoning [15, 16]. The protein binds claudin-4 and claudin-3 on targeted cells [17, 18]. The claudin family consists of at least 27 proteins that are essential for tight-junction formation in epithelial and endothelial cells and are important in controlling paracellular transport and the maintenance of cell polarity [19–23]. The binding of CPE to claudins triggers the formation of a multi-protein membrane pore complex, leading to a loss of cellular osmotic equilibrium and rapid cell lysis [24, 25]. Cells that lack claudin-3 or -4 expression are unaffected by the toxin [11, 17]. Numerous studies have shown that colon carcinoma and other epithelial tumors exhibit increased claudin-3 and/or -4 expression, suggesting that CPE might selectively target such tumors [26–37]. In our previous study we reported the successful tumor targeted in vitro and in vivo suicide gene therapy [11]. Based on this, the present approach is employing CPE gene therapy to selectively eradicate claudin-3 and -4 expressing colon carcinomas as a new strategy for this tumor entity.

Here, we use in vitro and in vivo approaches to demonstrate that claudin-3 and -4 expressing human colon cancers can be successfully treated by CPE gene transfer. CPE expression in these cells permits a rapid and selective eradication of colon cancer, further enhanced by a toxin-mediated bystander effect. We show that CPE specifically binds to the claudins in these cells and provide data on the kinetics of cytotoxicity as well as intracellular distribution of CPE after gene transfer. Our study reveals that CPE gene therapy can be used for the successful treatment of colon cancer.

## Methods

### Cell lines

Human SW480, SW620, HCT116 colon carcinoma and isogenic Sk-Mel5 and Sk-Mel5 Cldn-3-YFP melanoma cell lines were grown in RPMI medium (Gibco, Life technologies, Darmstadt, Germany), 10% FCS (Biochrom, Berlin,Germany). The colon carcinoma lines CaCo-2 and HT-29 were grown in DMEM (Gibco), 10% FCS (Biochrom). All lines were kept at 37 °C, 5% CO_2_. Claudin-3-YFP stably transfected cells were selected with 0.5–1.5 mgml^-1^ G418 (Gibco). The expression vector for Cldn-3-YFP is based on pEYFP-N1 [9]. The identity of all cell lines was confirmed by STR-genotyping (DSMZ, Braunschweig, Germany).

### Quantitative real-time RT-PCR

Cell lysis and isolation of total RNA was done using GeneMatrix Universal RNA Purification Kit EURx (Roboklon, Berlin, Germany). 50 ng of RNA was reverse transcribed and real-time RT-PCR (qRT-PCR) was performed with SYBR GREEN. Each real-time PCR (qPCR) was done using the LightCycler 480 (Roche Diagnostics, Mannheim, Germany). Following primers were used for claudin-3: forward 5’-CTGCTCTGCTGCTCGTGTCC-3’; reverse 5’-TTAGACGTAGTCCTTGCGGTCGTAG-3’; for claudin-4: forward 5’-CCTCTGCCAGACCCATATAA-3’; reverse 5’-CACCGTGAGTCAGGAGATAA-3’. The cycle conditions were as follows: 90 °C for 30 s, 95 °C for 5 s, 57 °C for 5 s and 72 °C for 10 s for 45 cycles. Normalization was done with the human housekeeping gene glucose-6-phosphate dehydrogenase (hG6PDH) using the hG6PDH Roche Kit (Roche Diagnostics).

### Western Blot

For protein analysis, cells or tissue cryosections were lysed in RIPA buffer (50 mM TRIS, 150 mM, NaCl, 1% Nonidet P-40, 0.5% sodium deoxycholate, protease inhibitor, ddH_2_O) and 25 μg of protein was electrophorezed in 10% precast NuPAGE gels (Invitrogen), 1 h at 180 V. The proteins were transferred to nitrocellulose membranes (Hybond-C Extra, Amersham, Freiburg, Germany) by semidry blotting (Turbo Blot BioRad, Munich, Germany) at 20 V, 25 min. Membranes were blocked for 1 h at room temperature (RT) in TBS (50 mM Tris, 150 mM NaCl, pH 7.5, 5% fat-free dry milk and 2.5% casein) and washed in TBST (0.05% Tween 20 in PBS), 2 x 5 min at RT. As primary antibody rabbit anti-claudin-3 antibody (1:3000, Acris, Herford, Germany), rabbit anti-claudin-4 antibody (1:3000, Acris), rabbit anti-CPE (1:4000, Acris), mouse monoclonal anti-β-tubulin (1:1000, BD Bioscience, MD, USA) or mouse monoclonal anti-β-actin antibody (1:10000, Sigma-Aldrich, MI, USA) was added respectively over night at 4 °C and washed in TBST. As secondary HRP-labeled goat anti-rabbit-IgG antibody (1:10000, Promega, Madison, WI, USA) or goat anti-mouse IgM/IgG (1:10000, Sigma-Aldrich) was added for 1 h, RT. Membranes were washed in TBST. Detection was done using ECL solution (Amersham) and exposure to Kodak X-Omat AR film (Kodak, Stuttgart, Germany).

### Clostridium perfringens enterotoxin (CPE) expressing plasmids

For transfection experiments the pCpG-optCPE (optCPE) plasmid was used [11]. For construction of plasmid encoding optCPE-GFP fusion protein, cDNA of CPE was amplified by PCR from optCPE and cloned into the Pst I site of pcDNA3.1/CT-GFP (GFP Fusion TOPO TA Expression Kit, Invitrogen Life Technology) resulting in pcDNA3-optCPE-GFP (optCPE-GFP). Preparation of plasmid-DNA was done using the Jetstar Plasmid Purification Maxi Kit (Genomed, Löhre, Germany).

### Transfection of human tumor cell lines

Cells were seeded into 6-well plates and transfected with 1.75–3.5 μg DNA (pCpG-optCPE, pCpG-mcsG2/pcDNA3.1 as empty vector or pcDNA3-optCPE-GFP fusion protein vector) using the transfection reagents Fugene X-treme (Roche), Fugene HD (Roche) or Metafectene (Biontex) as recommended by the manufacturer. To ensure comparable transfection rates, transfection efficiency for each cell line was determined by transfection of green fluorescent protein expressing plasmid pEGFP-N1 (Clontech, Mountain View, CA, USA) and analyzed using FACS Calibur (Becton Dickinson, San Jose, CA, USA). The number of green fluorescence protein expressing cells was quantified 48 h after transfection and given as a percentage of green fluorescent protein positive cells.

### RNAi

For knock-down experiments cells were seeded in 6-well plates and transfected with 50 nM siRNA control, siRNA Claudin-3 (iBONi siRNA box Cldn3 gene ID1363, Riboxx, Germany) or siRNA Claudin-4 (iBONi siRNA box Cldn4 gene ID1364, Riboxx, Germany) using Lipofectamine RNAiMax Reagent (Thermo Fisher Scientific, Darmstadt, Germany) according to the manufacturer’s instructions. Cells were seeded for recCPE treatment or optCPE gene transfer 48 h after siRNA transfection.

### MTT cytotoxicity assay

MTT assay was performed to test cytotoxicity of recombinant CPE or after optCPE transfection and biological activity of released CPE from transfected cells. For sensitivity testing of the cell lines towards recombinant CPE 6 × 10^3^- 4 × 10^5^ cells were seeded into 96-well plates and 24 h later the toxin was added at different concentrations (0, 50, 100, 150 ng ml^-1^) and incubated for 72 h. For determination of the biological activity of CPE in supernatants of transfected cells, 6 × 10^3^ non-transfected cells were seeded into 96-well plates. After 24 h, 100 μl of supernatants from optCPE transfected cells were added to the respective non-transfected cells and incubated for 72 h. For all cytotoxicity assays MTT (3-(4,5-dimethylthiazyol-2yl)-2,5-diphenyltetrazolium bromide (5 mg ml-1, Sigma) was added after 72 h of CPE incubation and absorbance was measured in triplicates at 560 nm in a microplate reader (Tecan, Groedig, Austria). Values are expressed as percentage of untreated controls.

### CPE ELISA

Ridascreen *Clostridium perfringens* Enterotoxin ELISA (R-Biopharm, Darmstadt, Germany) was performed to quantify CPE in supernatants 24 h or 48 h after transfection. For this, 4 × 10^5^ cells were seeded into 6-well plates and transfected with pCpG-optCPE or pCpG-mscG2 (e.v.) plasmid-DNA. Supernatants were used for the detection as recommended by the manufacturer. For analyzing potential shedding of CPE into the blood of animals, which received gene transfer, blood was collected and CPE was quantified in serum samples. Recombinant CPE was used as standard at serial dilutions of 0.4 ng to 25 ng CPE ml^-1^. Measurements were done in duplicates at 450 nm in the microplate reader (Tecan). Values are expressed as percentage of untreated controls.

### Immunofluorescence and immunohistochemistry

For immunofluorescence, 2 × 10^5^ cells were seeded onto cover slips (Steiner GmbH, Siegen Eiserfeld, Germany). After 24 h cells were treated with Hoechst 33342 (5 μM) or recombinant CPE (250 ng ml^-1^, R-Biopharm) or were transfected with pcDNA3.1 as empty vector, pCpG-optCPE (optCPE) or pcDNA3-optCPE-GFP (optCPE-GFP) as described. Cells were washed with PBS, fixed 15 min in 3.7% (v/v) formaldehyde in PBS, quenched 20 min with 0.1 M glycin in PBS and blocked 1 h with 1% (w/v) bovine serum albumin and 0.05% Tween 20 in PBS at RT. As primary antibody, rabbit anti-human claudin-3 or rabbit anti- human claudin-4 antibody (1:100, Acris) or rabbit anti-CPE (1:1000, Acris) was added for 2 h at RT. Cells were washed with TBST. Alexa 488 labeled goat anti-rabbit-IgG (1:1000, Invitrogen) or Alexa 555 labeled goat anti-rabbit-IgG (1:1000, Invitrogen) was added as secondary antibody for 1 h at RT. For staining of nuclei DAPI (Sigma-Aldrich) and for staining of the cytoplasm Alexa 555 phalloidin (Thermo Fisher Scientific) was used. Cells were evaluated in a fluorescence microscope (Zeiss, Jena, Germany).

For immunohistochemistry of the patient-derived xenotransplant (PDX, EPO GmbH, Berlin, Germany) tumor samples 3–5 μm paraffin embedded tumor sections were deparaffinized, fixed with 0.04% glutaraldehyde for 15 min at RT, quenched 20 min with 0.1 M glycin, incubated 10 min with 3% H_2_O_2_, washed with PBS, permeabilized by 0.2% Triton X-100 in PBS for 10 min, RT and blocked 1 h with 1% (w/v) bovine serum albumin and 0.05% Tween 20 in PBS at RT. Primary antibody rabbit anti-human claudin-3/-4 (1:200, Acris) was added for 2 h, RT. Sections were washed with PBS and as secondary antibody HRP-labeled goat anti-rabbit antibody (1:200, Promega) was added for 1 h, RT, then washed in PBS and incubated with diamino-benzidine (DAB, DAKO, Hamburg, Germany) 1 min, at RT, washed in PBS, counterstained for 30 s with hemalum (Roth, Karlsruhe, Germany), rinsed in water, covered with glycergel (DAKO) and evaluated in a light microscope (Zeiss).

### in vivo optCPE gene transfer

For establishment of subcutaneous tumors, pieces of app. 3 × 3 mm in size of patient derived colorectal cancer xenograft tissue (PDX, Co7515*, lung metastasis of colon cancer, see Additional file 1) were inoclulated into the left flank of female NMRI: nu/nu mice (*n* = 5 animals per group). When tumors reached a mean volume of 0.3 cm^3^, animals were randomized into treatment groups. Intratumoral non-viral optCPE gene transfer was performed in anesthetized animals by jet-injection. Therefore, 50 μg plasmid DNA of respective vector construct was applied by 5 injections (jet injector, EMS Medical Systems SA, Nyon, Switzerland) of 10 μl injection volume (1 μg DNA μl^-1^ PBS). The in vivo optCPE gene transfer gene transfer was performed once at day 46 post tumor inoculation.

Tumor volumes (TV) were measured at indicated time points and calculated using the formula: TV = (width^2^ x length)/2. As toxicity parameters body weight, clinical signs and behavior were recorded for all mice twice a week. Animals were sacrificed and tumors were harvested for further analysis.

### Statistical analysis

For statistical analyses of the in vitro experiments the Student’s t-test, 1way-ANOVA test and 2way-ANOVA test was used. For the analyses of in vivo optCPE gene transfer experiments the non-parametric Mann-Whitney test was used. Error values for the in vitro experiments are S.D. and for in vivo optCPE gene transfer experiment S.E.M.

## Results

### Claudin-3 and -4 expression in human colon carcinoma cell lines

**Fig. 1:**
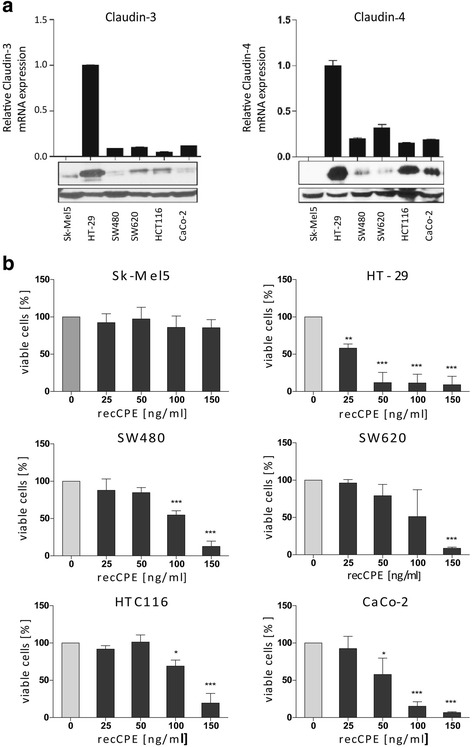
Expression analysis and sensitivity testing of human colon carcinoma cell lines. Colon cancer cell line HT-29, SW480, SW620, HCT116 and CaCo-2 and human melanoma cell line Sk-Mel5 were characterized. **a** Quantitative real-time RT-PCR and western blot for claudin-3 (*left*) and claudin-4 expression (*right*), revealing high claudin-3 and -4 expression in HT-29 and HCT116, mediate claudin-3 expression in SW480 and SW620 and a strong claudin-3 expression in CaCo-2 and no claudin-3 or -4 expression in Sk-Mel5 cells. Columns: mean of triplicates, bars: SD. **b** Cell sensitivity toward recombinant CPE protein. Tumor cells were treated at indicated concentrations for 72 h. The cytotoxicity was determined by MTT assay and compared to untreated control. Strong cytotoxic effects were seen in all colon cancer cells. Claudin-negative cell line Sk-Mel5 was unaffected and used as negative control. All assays were performed in three independent experiments and expressed as mean percent of untreated control. Bars: SD. Level of significance was calculated by Student’s t-test; * *P* < 0.05, **; *P* < 0.001, *** *P* < 0.0001

CPE toxicity is dependent on the presence of claudin-3 and -4. Therefore, we tested human colon carcinoma cell lines HT-29, SW480, SW620, HCT116 and CaCo-2 and the human melanoma cell line Sk-Mel5 for claudin expression. We found a high expression of claudin-3 and -4 in HT-29 cells at protein- and mRNA-level. SW620 and HCT116 cells revealed a lower claudin-3 and -4 expression, whereas SW480 and CaCo-2 cells expressed claudin-4 at a lower level (Fig. 1a). The melanoma line Sk-Mel5 was negative for claudin-3 and -4 expression and was used as negative control in all in vitro experiments.

### Sensitivity of human colon carcinoma cells towards recombinant CPE

The cell lines HT-29, SW480, SW620, HCT116, CaCo-2 and Sk-Mel5 were treated with recombinant CPE (recCPE) for sensitivity testing. The treatment revealed a strong correlation between claudin-3 and -4 expression and sensitivity towards recCPE (Fig. 1b). The high claudin-3 and -4 expressing cell line HT-29 showed highest sensitivity to recCPE with 88% toxicity at a concentration of 50 ng ml^-1^, supported by the low EC_50_ value of 26.1 ng CPE ml^-1^ (Table 1). CaCo-2 cells were less sensitive than HT-29, but still showed a low EC_50_ value of 54 ng CPE ml^-1^ compared to the other colon carcinoma cells. Although HCT116, SW620 and SW480 cells have comparable claudin-3 and -4 expression, these cells were less sensitive to the recCPE treatment, reflected by EC_50_ values of about 100 ng ml^-1^. However, concentration of 150 ng CPE ml^-1^ was sufficient to kill 80-90% of these cells. By contrast, the claudin-negative Sk-Mel5 cells were insensitive towards the toxin, supporting that CPE toxicity is restricted to claudin-3 and -4 expressing cells.

**Table 1:** EC_50_ values of recombinant CPE

*Cell line*	*recCPE* *EC*_*50*_ *[* *ng ml* ^-1^ *]*
Sk-Mel5	-
HCT116	102.7
SW620	102.5
SW480	101.7
CaCo-2	54.18
HT-29	26.1

### In vitro CPE expression and selective cytotoxicity after CPE gene transfer

**Fig. 2:**
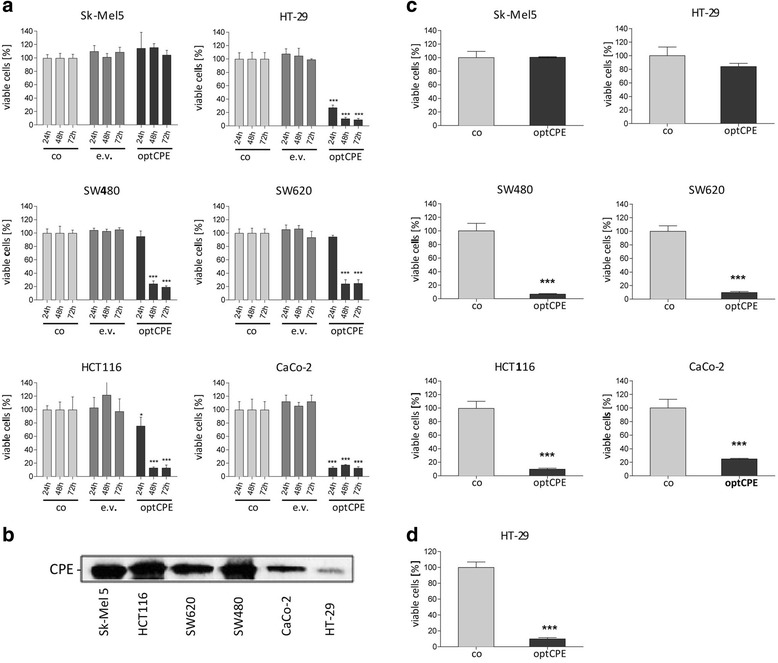
Cytotoxicity of CPE gene transfer in human colon carcinoma cells. **a** Time-dependent cytotoxicity of CPE after transfection with the pCpG-optCPE (optCPE) and empty vector (e.v.). MTT cytotoxicity assay was performed 24, 48 and 72 h after transfection. All transfected colon cancer cells reveal strong cytotoxic effects (*P* < 0.0001) mediated by optCPE 48 h after gene transfer. Sk-Mel5 cells were unaffected. Measurements were done in triplicates and expressed as mean percent of untreated control. Bars: SD. Level of significance was calculated by 2way-ANOVA; * *P* < 0.05, ** *P* < 0.001, *** *P* < 0.0001. **b** Analysis of CPE release by optCPE transfected human colon carcinoma cell lines. Media of optCPE transfected cells were harvested 48 h after transfection and analyzed by western blot. Representative blot shows released CPE within the media in all transfected cells. **c** Analysis of biological activity of released optCPE. optCPE containing media were added once to respective non-transfected cell lines and cytotoxicity was determined by MTT 72 h after application. SW620, SW480, HCT116 and CaCo-2 cells showed strong significant decrease in cell viability, correlating with amount of released optCPE (see **b**). HT-29 cells released low amounts of optCPE protein (see **b**) after transfection and cell viability was not reduced after application of optCPE containing medium. Columns represent mean percentage of untreated control cells. Bars, SD. Measurements were performed in triplicates and levels of significance were calculated by Student’s t- test; *** *P* < 0.0001. **d** Analysis of cytotoxic effect after repeated application of CPE containing medium in HT-29 cells. optCPE containing medium of optCPE transfected cells (see **b**) was added twice a day at 5.4 ng CPE ml^-1^ (see Table 3) to non-transfected HT-29 cells. MTT assay was performed 72 h after treatment and significant decrease of cell viability (*P* < 0.0001) was measured. Measurements were done in triplicates and expressed as mean percent of untreated control. Bars: SD. Level of significance was calculated by Student’s t-test; *** *P* < 0.0001

To evaluate the therapeutic potential of CPE gene transfer HT-29, HCT116, SW480, SW620, CaCo-2 and Sk-Mel5 cells were transfected with either the optimized pCpG-optCPE expressing vector (optCPE) or the pCpG empty vector (e.v.). Cytotoxicity was determined 24 h, 48 h and 72 h after transfection by MTT assay. The experiments showed high toxicity of optCPE expressing vector in all claudin-positive cells 48 h after transfection with toxicity rates of 76–92% (Fig. 2a). In HT-29 and CaCo-2 cells optCPE exerted 70–87% cytotoxicity as soon as 24 h after transfection. In SW480, SW620 and HCT116 cells however, significant (*P* < 0.001) reduction of cell viability was detectable after 48 h. By contrast, optCPE transfection did not have any impact on the claudin-negative melanoma Sk-Mel5 cells, indicating strict claudin-selectivity of CPE after gene transfer.

These data further support the correlation between claudin-3 and -4 expression and CPE mediated toxicity.

### Bystander effect of CPE gene transfer

The transfection experiments revealed a much higher CPE toxicity than it would have been anticipated by transfection rates of 35–75% for the cell lines (Table 2). The bystander effect, which is mediated by released optCPE, could explain why also non-transfected colon carcinoma cells are affected after treatment. To prove this, we analyzed the medium of optCPE transfected cells. Western blot analysis (Fig. 2b) and quantification by CPE-ELISA (Table 3) confirmed the presence of optCPE in the medium 48 h after transfection, ranging from up to 5.4 ng ml^-1^ released CPE by HT-29 cells to up to 231.67 ng ml^-1^ CPE liberated by HCT116 cells. The biological activity of released CPE was tested by adding the medium, collected 48 h after transfection, to respective non-transfected cells (Fig. 2c). The cytotoxicity assay showed up to >90% toxicity in HCT116, SW480 and SW620 cells and about 76% in CaCo-2 cells, with no effect on claudin-3 and -4 negative Sk-Mel5 cells. However, addition of optCPE containing medium to respective HT-29 cells only caused minor toxicity, due to the single application of CPE at low concentration of only 5 ng CPE ml^-1^. For a better mimesis of the conditions achieved by gene transfer, this medium was added twice a day to respective non-transfected HT-29 cells (Fig. 2d), since in transfected HT-29 cells much higher toxicity was seen. This demonstrated that repeated application, even at low CPE concentrations, is sufficient to achieve cytotoxicity, which is comparable to optCPE-transfected HT-29 cells (Fig. 2a). These data again support the efficiency of optCPE gene therapy by prolonged presence of the toxin and the associated bystander effect for colon cancer eradication.

**Table 2:** Transfection efficiency of the human cancer cell lines, determined by transfer of the pEGFP vector and FACScan

*Cell line*	*Transfection efficiency (% GFP-positive cells)*
Sk-Mel5	48.6%
HCT116	75.4%
SW620	35.2%
SW480	52.6%
CaCo-2	55.5%
HT-29	48.2%

**Table 3:** Quantification of CPE release into the cell culture medium of empty vector or optCPE-transfected human colon carcinoma cells (error values are S.D.)

*Cell line*	*Vector*	*CPE ± SD* *[ng ml* ^*-1*^ *]*
Sk-Mel5	e.v.	0
	optCPE	46.92 ± 22.06
HCT116	e.v.	0
	optCPE	231.67 ± 46.21
SW620	e.v.	0
	optCPE	67.30 ± 1.33
SW480	e.v.	0
	optCPE	80.51 ± 5.97
CaCo-2	e.v.	0
	optCPE	16.50 ± 3.15
HT-29	e.v.	0
	optCPE	5.40 ± 0.9

### Claudin-specificity and analysis of claudin-3/optCPE-GFP co-localization

**Fig. 3:**
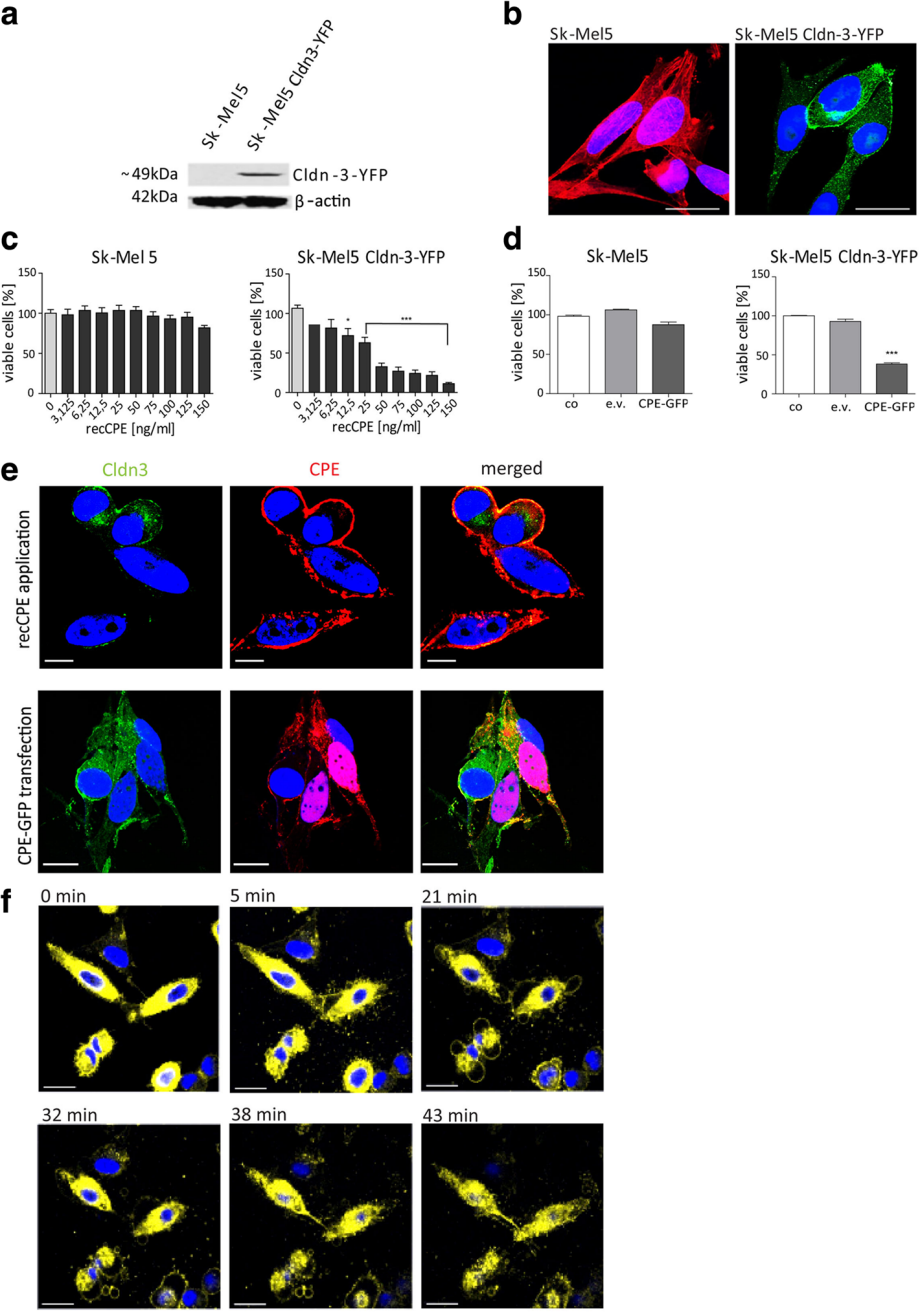
Specificity of CPE in the isogenic cell lines SK-Mel5 and SK-Mel5 Claudin-3-YFP **a** Western blot analysis for claudin-3-YFP gene expression in Sk-Mel5 Cldn-3-YFP cells and no claudin-3 expression in Sk-Mel5 cells. **b** Representative immunofluorescence of Sk-Mel5, and Sk-Mel5 Cldn-3-YFP cells. Claudin-3 negative cells were counterstained with Phalloidin (*red*). Nuclei were counterstained with Hoechst33343. Scale bar: 10 μm. The image demonstrates specific claudin-3 expression (*yellow*) of Sk-Mel5 Cldn3-YFP cells. **c** Isogenic Sk-Mel5 and Sk-Mel5 Cldn-3-YFP cells were treated with recCPE for 72 h at indicated concentrations. recCPE toxicity was determined by MTT cytotoxicity assay. Decreased cell viability mediated by recCPE cytotoxicity is demonstrated in Sk-Mel5 Cldn-3-YFP cells, SK-Mel5 cells remained unaffected. All assays were performed in three independent experiments and expressed as mean percent of untreated control. Bars: SD. Level of significance was calculated by 2way-ANOVA; * *P* < 0.05, **; *P* < 0.001, *** *P* < 0.0001. The assay revealed highest specificity of recCPE mediated cytotoxicity by binding to claudin-3. **d** Cytotoxicity of optCPE-GFP gene transfer in isogenic Sk-Mel5 pair and proof of claudin specificity. Isogenic cells were transfected with empty vector (e.v.) expressing or optCPE-GFP expressing construct. MTT assay was performed 72 h after transfection. Controls are transfection reagent and e.v. transfected cells. All assays were performed in three independent experiments and expressed as mean percent of untreated control. Bars: SD. Level of significance was calculated by 1way-ANOVA, *** *P* < 0.0001. The assay demonstrates high cytotoxicity for optCPE-GFP expressing Sk-Mel5 Cldn3-YFP cells, which acts selectively on claudin-3 cells, leaving claudin-negative Sk-Mel5 cells unaffected. **e** Representative images for co-localization of Cldn-3-YFP and CPE**.** Upper panel represents Sk-Mel5 Cldn-3-YFP cells, incubated with recCPE (250 ng ml ^-1^) for 30 min and counterstained with specific CPE antibody and Alexa555 labeled secondary antibody; Cldn-3-YFP (*green*), recCPE (*red*) and co-localization of CPE/Cldn-3-YFP (*yellow*) is shown. Nuclei were counterstained with Hoechst 33343 (*blue*). Scale bar: 10 μm. Lower panel demonstrates Sk-Mel5 Cldn-3-YFP cells transfected with optCPE–GFP and fixed 24 h later. Expressed Cldn-3-YFP (*green*), optCPE (*red*) and their co-localization (*yellow*) were detected (*indicated by arrows*). CPE was mainly located at the cell membrane. Counterstaining of nuclei with Hoechst 33343 (*blue*). Scale bar: 20 μm. These images confirm co-localization and specificity of CPE binding to claudin-3 as well as CPE accumulation within the cytoplasm. **f** Time-dependent cell death analysis after external application of recCPE (150 ng ml ^-1^). Sk-Mel5 Cldn-3-YFP cells were imaged for 45 min after recCPE addition. This process continued with swelling of treated cells, cell blebbing and size reduction of nuclei. Cell nuclei were stained with Hoechst33343 (*blue*). Scale bar: 25 μm

To elucidate claudin-specificity of CPE activity, the optCPE-GFP fusion protein was used for treatment of an isogenic pair of claudin-3 negative and claudin-3 positive Sk-Mel5 cells. They were generated by transfection of the claudin-3-YFP (Cldn-3-YFP) expressing vector. The Cldn-3-YFP expression in Sk-Mel5 cells and membranous localization was verified by Western blot analysis and fluorescence microscopy (Fig. 3a, b). Both show distinct membranous localization of claudin-3, notably within cell-cell-contact regions. The isogenic pair was first treated with recCPE for 72 h (3.125–150 ng CPE ml^-1^), leading to >90% cytotoxicity in claudin-3 expressing Sk-Mel5 cells, whereas the claudin-3 negative cells remained insensitive (Fig. 3c). For further validation of claudin-specificity, cells were transfected with optCPE-GFP fusion protein. Strong cytotoxic effect, resulting in 62% reduction of cell viability, was measured 72 h after optCPE-GFP gene transfer (Fig. 3d).

In addition to the previous described knock-in experiments, we also performed knock-down experiments in the human colon cancer cell lines HCT116 and SW480 by using short interfering RNA, targeting claudin-3 and -4 to proof the important role of the claudins in the CPE mediated effect. The down-regulation of both claudins (siCldn3 + siCldn4) led to a significantly reduced responsiveness of the colon cancer cells towards recCPE treatment and optCPE gene transfer compared to control (siCo) treated cells, demonstrating specific activity of CPE to its receptors claudin-3 and -4 (Additional file 2).

To prove the specific co-localization of CPE and claudin-3, we analyzed Cldn-3-YFP expressing Sk-Mel5 cells after treatment with recCPE or after transfection with the optCPE-GFP expressing vector by confocal fluorescence microscopy. After external application of recCPE, binding of the toxin in areas of strong Cldn-3-YFP expression within the cell membrane and cytoplasm was observed (Fig. 3e *upper panel*). Analysis of CPE-distribution after transfection with optCPE-GFP fusion protein expressing vector revealed a membranous binding of CPE and cytoplasmic CPE accumulation (Fig. 3e *lower panel*).

**Fig. 4:**
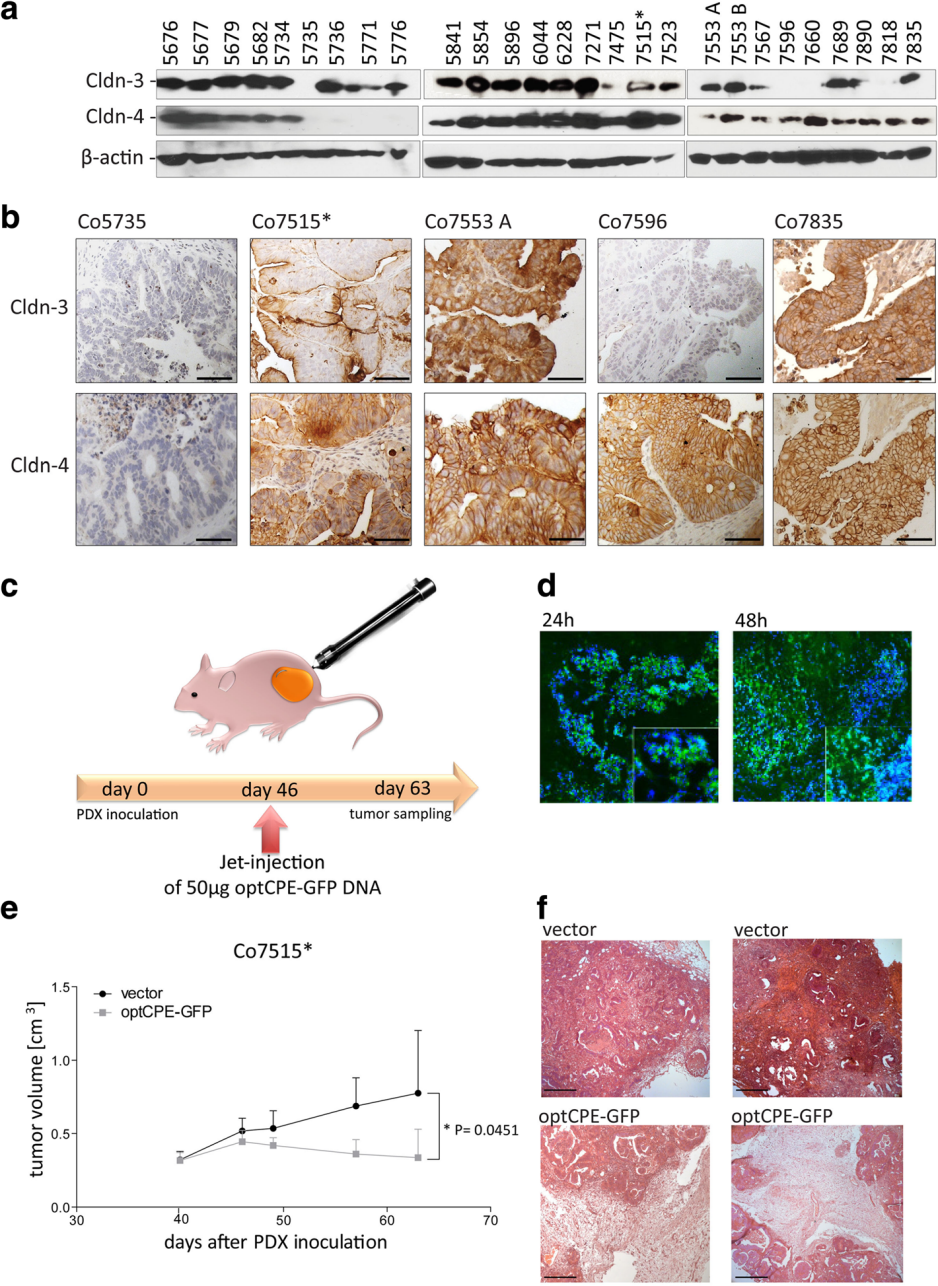
Analysis of claudin-3 and -4 expression and CPE sensitivity in patient derived colon carcinoma xenografts. **a** Western blot analysis for claudin-3 and claudin-4 expression, demonstrating highest expression of the claudins in colon cancer, since 26 of 27 analyzed tumors expressed claudin-3 and/or -4. Only one tumor (Co5735) did not express one of the claudins. **b** Representative images of immunohistochemistry for claudin-3 and -4 expression (brown staining) in the PDX tumors Co5735, Co7515*, Co7553A, Co7596 and Co7835, which correlated with protein expression (see *A*). The claudin-negative PDX Co5735 did not show any specific staining, whereas membranous localization of claudin-3 and/or -4 was found within the other representative PDX models. **c** Representative scheme of non-viral in vivo gene transfer using the jet-injection. **d** Intratumoral distribution of optCPE-GFP fusion protein expression 24 and 48 h *after* in vivo gene transfer of Co7515* tumors. Representative immunofluorescence images revealed strong optCPE-GFP accumulation within the tissues (*green*). Tissue disruption and cell death can be already seen after 24 h but strong areas of necrosis where detected after 48 h (see insets), indicating a successful gene transfer. Cell nuclei were counterstained with Dapi (*blue*). Scale bar: 100 μm. **e** Inhibition of Co7515* PDX tumor growth after optCPE-GFP gene transfer. The vector-transfected tumors serve as control. The in vivo optCPE-GFP gene transfer led to significant reduction in tumor growth (*P* = 0.0451); level of significance was determined by using non-parametric t-test; bars, S.E.M. **f** Representative H&E staining of terminal tumor sections (day 63 after Co7515* PDX inoculation) show incidence of massive necrotic areas in optCPE-GFP transfected tumors compared to vector control, indicating applicability of this gene therapy for colon carcinoma. Scale bar: 100 μm

### Time lapse of CPE toxicity

Time lapse evaluation after recCPE treatment by live fluorescence microscopy in Sk-Mel5 Cldn-3-YFP cells revealed a rapid process of cell death starting at 20 min after recCPE addition. This process continued with swelling of treated cells, cell blebbing and size reduction of nuclei (Fig. 3f). To quantify the decrease in size nuclei area was measured over a time period of 45 min. These measurements showed significant (***P < 0.001) size reduction of 50%, indicative for rapid nuclear damage and cell death (Additional file 3).

### Claudin-3 and -4 expression in patient-derived colon carcinoma xenografts

To extend the approach beyond the established in vitro models to in vivo optCPE gene transfer application, claudin-3 and -4 expression was analyzed in human patient-derived colon carcinoma xenografts (PDX) at protein level (Fig. 4a). We found claudin-3 and/or -4 expression in 26 of 27 analyzed tumors of which 19 showed high expression of both proteins. Only one tumor (Co5735) did not express claudin-3 or -4. The immunohistochemistry confirmed Western blot data and indicates mostly membranous localization as shown for representative PDX tumors (Fig. 4b).

### In vivo expression of CPE and antitumoral efficiency after non-viral intratumoral CPE gene transfer

The intratumoral CPE expression was first analyzed after non-viral intratumoral in vivo optCPE gene transfer jet injection of the optCPE-GFP expressing vector (Fig. 4c). Efficient expression of optCPE-GFP fusion protein was determined 24 h and 48 h after gene transfer. The immunofluorescence revealed areas of strong optCPE-GFP expression within tumor tissues (Fig. 4d), indicating a successful in vivo optCPE gene transfer.

After optCPE-GFP jet-injection gene transfer (day 46 after tumor implantation), significant antitumoral activity by optCPE-GFP mediated cytotoxicity was observed in transfected Co7515 PDX-bearing mice. Tumor growth inhibition, as measured by tumor volume, was significant (*P = 0.0451) in optCPE-GFP transfected tumors compared to vector transfected control group (Fig. 4e). Evaluation of HE-stained sections of optCPE transfected tumors revealed massive necrotic areas within the tumors due to optCPE action (Fig. 4f). In all animals no systemic toxicities, such as body weight loss, diarrhea or increase in body temperature were observed, which strongly indicates safety of this gene therapeutic approach (Additional file 4). This is supported by the fact, that analysis for potential appearance of CPE in the blood of the animals by ELISA revealed no shedding of the toxin to the circulation (Additional file 5).

## Discussion

### Pore-forming CPE as effective cancer therapeutic

Genes coding for bacterial toxins have shown their therapeutic potential for cancer treatment in cancer gene therapy [10, 37–40]. Especially pore-forming toxins like Streptolysin O from *Streptococcus pyogenes* and Clostridium perfringens enterotoxin (CPE) from *Clostridium perfringens* are of increasing interest, of which CPE possesses high specificity in action [12, 13, 41]. The targeted action of CPE is mediated by binding to claudin-3 and -4, which are frequently overexpressed in cancers. This is in line with other studies, which show an upregulation of claudin-3 and -4 in colorectal cancers compared to their expression in normal mucosa [26, 29]. In fact these findings render claudin-3 and -4 an attractive target for selective therapy. In this study we demonstrated, that colon cancer cells also overexpress claudin-3 and-4, which therefore represents the potential target for CPE therapy.

CPE binding to the claudins results in rapid cell lysis [42, 43]. The antitumoral effect of recombinant CPE (recCPE) has been shown in several in vitro and in vivo studies. It was demonstrated however, that the therapeutic use of recCPE is restricted by the need of repeated application to achieve an antitumoral effect [29, 44–47].

This limitation can be overcome by gene therapeutic use of CPE-expressing vectors for improved and prolonged toxin action to treat cancers such as colon carcinoma [11, 18, 45].

### Selectivity of CPE-mediated rapid cytotoxicity

In this approach we demonstrated for the first time that CPE was efficiently expressed in colon cancer cells in vitro as well as in colon cancer PDX in vivo optCPE gene transfer, which led to effective tumor cell lyses in claudin-3 and -4 positive cells, whereas claudin-negative cells remained unaffected [48]. Several publications have characterized claudin-3 and -4 as natural and specific receptors for CPE [49–52]. Here, sensitivity of colon cancer cells towards external application of recCPE and more importantly of gene transfected CPE was shown. We demonstrated a direct correlation of claudinexpression, localization and CPE toxicity.

There is only one report about the use of CPE for cancer gene therapy [11], whereas other bacterial toxins, such as Pseudomonas exotoxin, Diphteria toxin and Streptolysin O, have already been analyzed regarding their gene therapeutic use and antitumoral effects. After transfection of Streptolysin O and Diphteria toxin expressing vectors, both toxins exerted toxic effects in vitro and in vivo, but also led to side effects, as these toxins act on non-tumor targets [8, 9, 12]. By contrast, CPE has the advantage of specific binding to claudin-3 and -4, which is accessible at the cell surface and potentially also in the cytoplasm of cancer cells. Previously we established a translation optimized CPE vector (optCPE), which combines both, target specificity and efficient cytotoxicity [11]. Using the optCPE and optCPE-GFP fusion protein in this approach, rapid tumor destruction in vitro and in vivo was achieved after optCPE gene transfer. In fact, the selectivity of CPE tumor cell eradication was further supported by the use of the isogenic Sk-Mel5 cells, in which only the claudin-3 expressing cells were susceptible towards CPE action and claudin negative cells remained unaffected. More importantly, siRNA-mediated down-regulation of claudin-3 and -4 in colon cancer cells led to decreased responsiveness of these cells towards CPE toxicity. This strengthens the importance of the claudins for targeted action of CPE in colon cancer.

### Release of CPE and bystander effect

It is still unknown how intracellularly expressed CPE is released to bind the extracellular domains of the claudins. CPE is naturally produced by the clostridium bacteria and only liberated by lyses during bacterial sporulation [53, 54]. As we have described previously and observed in this study, released CPE is present in the media of all transfected cells, suggesting that it is liberated independently of CPE-mediated cytotoxicity or claudin-3 and -4 expression, as it was also released by the claudin-negative Sk-Mel5 cells [11]. This supports the concept of the bystander effect, as it contributes to the efficiency of gene therapy.

The bystander effect was seen during in vitro optCPE gene transfer, as it eradicated up to 92% of colon carcinoma cells, although transfection efficiencies range only between 35 to 75%. The biological activity was further proven as non-transfected cells treated with released CPE containing media revealed strong cytotoxicity. Such effect can be crucial for improved therapeutic efficiency, particularly for in vivo optCPE gene transfer.

### Time lapse of CPE toxicity

Additionally we demonstrated the process of cell death after external application of recCPE by localization analysis and confirmed biological activity of released optCPE, as cell death was initiated as early as 20 min after treatment. Furthermore, this was validated by significant decrease of nuclear size by 50%, which could be caused by aggregation of chromatin during apoptosis, by chromatin dissolution or nuclear fragmentation during necrosis. In previous analyses for external therapeutic application of recCPE both, necrosis and apoptosis were reported, depending on the CPE doses used [25]. We further showed in our previous study that the intracellular accumulation of CPE induced rapid release of cytoplasmic lactate dehydrogenase (LDH), activation of caspases 3/7 in vitro and caused in vivo necrosis in CPE transfected tumors [11].

### CPE sensitivity of claudin-3 and -4 expressing patient derived colon carcinoma xenografts (PDX)

The selective and efficient cytotoxic effect of CPE gene transfer was proven for human colon cancer cell lines in vitro. Our study extended the promising anticancer potential of CPE gene therapy to in vivo optCPE gene transfer scrutinization in a patient derived subcutaneous xenograft model derived from a metastatic colorectal tumor. By screening of 27 colon carcinoma PDX for claudin-3 and/or -4, the clinical relevance of this approach was further supported, as 20 out of 27 PDX models showed both, claudin-3 and claudin-4 expression, whereas 3 revealed the expression of only claudin-3, suggesting, that binding of CPE can occur. We further demonstrated the selective and efficient antitumoral action of CPE gene therapy in the claudin-3 and -4 overexpressing PDX Co7515, where gross tumor necrosis of the treated tumors was observed. This strongly indicates the applicability of this suicidal gene therapy for the treatment of colon carcinomas.

## Conclusions

We report for the first time the successful tumor-targeted CPE gene therapy for colon carcinoma in vitro and in vivo optCPE gene transfer. This approach demonstrates that CPE gene transfer could be a promising and efficient option for a targeted suicide gene therapy of claudin-3 and/or -4 expressing colon carcinoma.

The CPE gene therapy is of particular interest if applied locally to treat either unresectable residual cancer tissue or to treat unresectable or refractory liver or lung metastasis of colorectal cancers. In fact, we did use in our in vivo optCPE gene transfer experiment the Co7515 PDX model, which highly expresses claudin-3 and -4 and is derived from lung metastasis of colon cancer. This provides some hint that such approach might be of value for the local control of the disease. Therefore, this strategy could be of particular value for treatment of therapy-refractory tumors or metastases thereof.

## Additional files

Additional file 1: Table S1. List of patient derived colon carcinoma xenografts (PDX) and their primary origin. (XLSX 39 kb)
Additional file 2: Figure S1. Knockdown of claudin-3 and -4 leads to reduced CPE activity in human colon cancer cells. **a** Sequences of used short interfering RNA (siRNA) targeting claudin-3 and -4. **b** Western blot analysis for claudin-3 and claudin-4 gene expression in human colon cancer cell lines SW480 (left panel) and HCT116 (right panel) 72 h after siRNA treatment, showing an efficient down-regulation of both with two independent siRNA compared to control (siCo). **c** Specific toxin responsiveness of claudin-3 and -4 down-regulated colon cancer cells. 72 h after siRNA transfection tumor cells were treated with recCPE at indicated concentrations for another 72 h. The cytotoxicity was determined by MTT assay and compared to siCo treated cells. A significantly reduced responsiveness (*** *P* < 0.0001) was demonstrated in both colon cancer cell lines, SW480 (left panel) and HCT116 (right panel). All assays were performed in two independent experiments and are expressed as mean percent of untreated control. Bars: SD. Level of significance was calculated by 2way-ANOVA (Bonferroni posttest). **b** Cytotoxicity of optCPE gene transfer in siRNA treated colon cancer cells and proof of claudin specificity. The siCldn3 + siCldn4 treated SW480 and HCT116 cells were transfected with optCPE construct 72 h after siRNA treatment. MTT assay was performed 72 h after CPE treatment and a significantly reduced CPE mediated cytotoxicity was observed in down-regulated SW480 (left panel) and also in HCT116 (right panel) cells compared to siCo treated cells. All assays were performed in two independent experiments and expressed as survival in optical density [OD]. Bars: SD. Level of significance was calculated by nonparametric, unpaired students t-test, *** *P* < 0.0001. Both assays demonstrate high selectivity of CPE on claudin-3 and -4 as down-regulated cells remain unaffected. (JPG 600 kb)
Additional file 3: Figure S2. Quantification of change in nuclear size by CPE treatment. Size of nuclei was measured after recCPE application over a time period of 45 min. The data show significant reduction in nuclear size, indicating rapid cell death mediated by recCPE. Area of three nuclei were calculated every 5 min by Imaris Cell 7.6. Value represents mean, errors are given as S.D. (JPG 111 kb)
Additional file 4: Figure S3. Influence of optCPE in vivo gene transfer on body weight. Body weight of Co7515* PDX bearing mice was measured during tumor growth inhibition. In all animals no systemic toxicities, such as body weight loss, were observed, which strongly indicates the safety of this gene therapeutic approach. (JPG 283 kb)
Additional file 5: Table S2. Quantification of CPE shedding into blood circulation of control-, vector- or optCPE-treated animals. (XLSX 38 kb)

## Abbreviations

Cldn: Claudin
CPE: *Clostridium perfringens* enterotoxin
e.v.: pCpG-mscG2 empty vector/pcDNA3.1 empty vector
optCPE: pCpG-optCPE expressing vector
optCPE-GFP: pcDNA3.1-optCPE-GFP expressing vector
PDX: patient derived xenograft
recCPE: recombinant CPE
