# Supplementary figures and images for: Rapid eradication of colon carcinoma by Clostridium perfringens Enterotoxin suicidal gene therapy
Source: BMC Cancer. 2017 Feb 13;17:129. doi: 10.1186/s12885-017-3123-x (PMC5307849; doi:10.1186/s12885-017-3123-x)

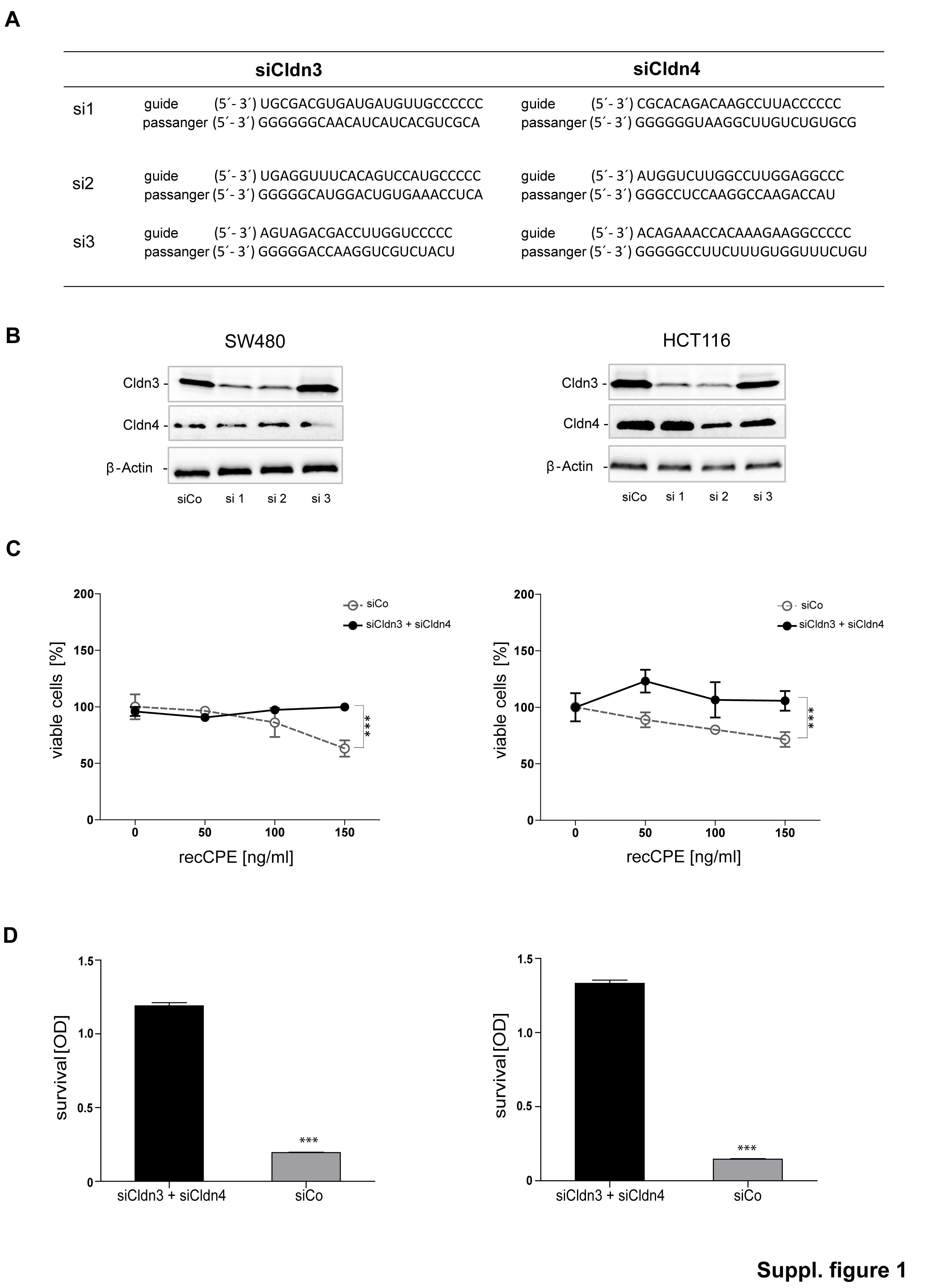

Supplement: Additional file 2: Figure S1. — Knockdown of claudin-3 and -4 leads to reduced CPE activity in human colon cancer cells. a Sequences of used short interfering RNA (siRNA) targeting claudin-3 and -4. b Western blot analysis for claudin-3 and claudin-4 gene expression in human colon cancer cell lines SW480 (left panel) and HCT116 (right panel) 72 h after siRNA treatment, showing an efficient down-regulation of both with two independent siRNA compared to control (siCo). c Specific toxin responsiveness of claudin-3 and -4 down-regulated colon cancer cells. 72 h after siRNA transfection tumor cells were treated with recCPE at indicated concentrations for another 72 h. The cytotoxicity was determined by MTT assay and compared to siCo treated cells. A significantly reduced responsiveness (*** P < 0.0001) was demonstrated in both colon cancer cell lines, SW480 (left panel) and HCT116 (right panel). All assays were performed in two independent experiments and are expressed as mean percent of untreated control. Bars: SD. Level of significance was calculated by 2way-ANOVA (Bonferroni posttest). b Cytotoxicity of optCPE gene transfer in siRNA treated colon cancer cells and proof of claudin specificity. The siCldn3 + siCldn4 treated SW480 and HCT116 cells were transfected with optCPE construct 72 h after siRNA treatment. MTT assay was performed 72 h after CPE treatment and a significantly reduced CPE mediated cytotoxicity was observed in down-regulated SW480 (left panel) and also in HCT116 (right panel) cells compared to siCo treated cells. All assays were performed in two independent experiments and expressed as survival in optical density [OD]. Bars: SD. Level of significance was calculated by nonparametric, unpaired students t-test, *** P < 0.0001. Both assays demonstrate high selectivity of CPE on claudin-3 and -4 as down-regulated cells remain unaffected. (JPG 600 kb) [file 12885_2017_3123_MOESM2_ESM.jpg]

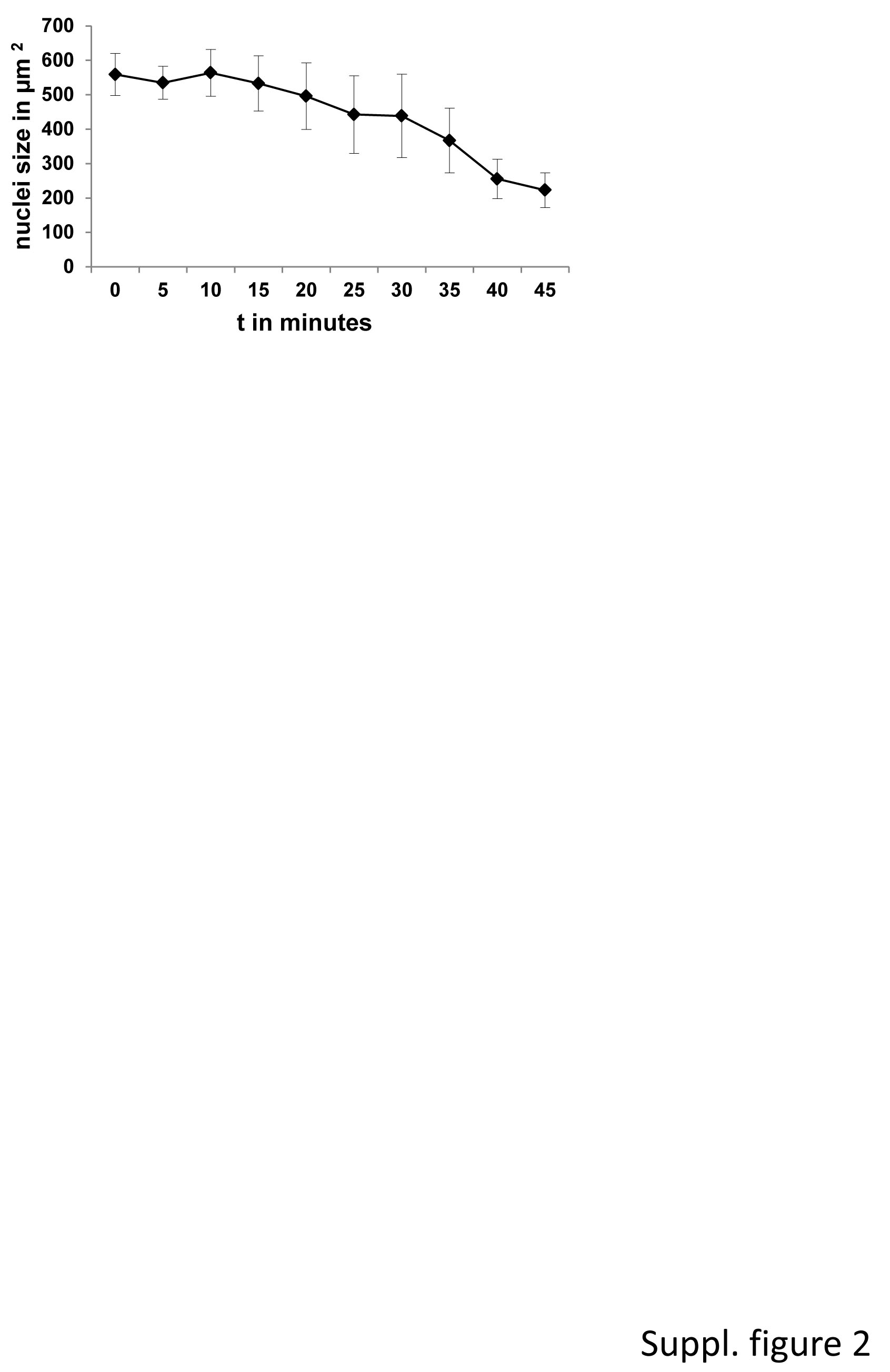

Supplement: Additional file 3: Figure S2. — Quantification of change in nuclear size by CPE treatment. Size of nuclei was measured after recCPE application over a time period of 45 min. The data show significant reduction in nuclear size, indicating rapid cell death mediated by recCPE. Area of three nuclei were calculated every 5 min by Imaris Cell 7.6. Value represents mean, errors are given as S.D. (JPG 111 kb) [file 12885_2017_3123_MOESM3_ESM.jpg]

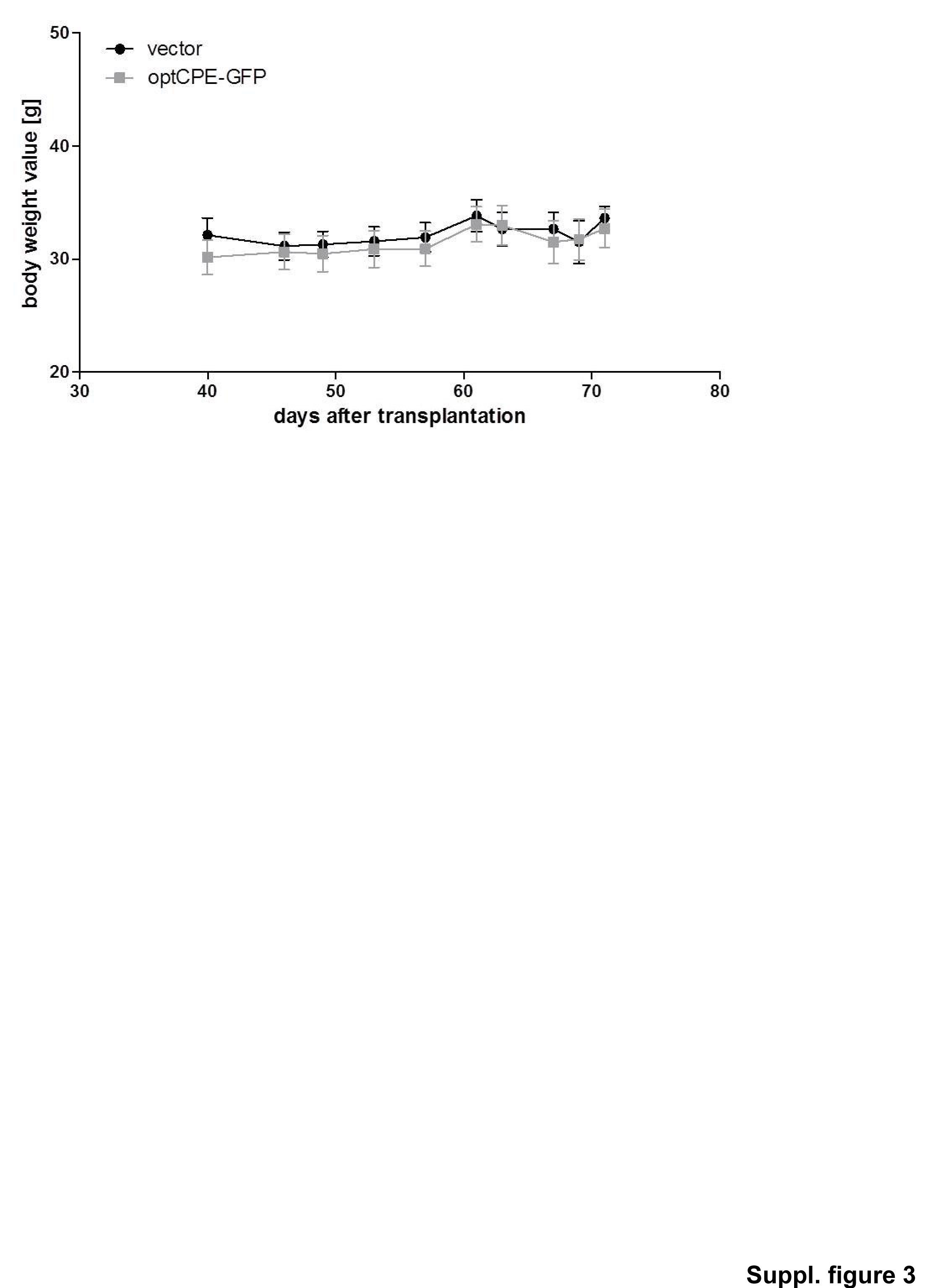

Supplement: Additional file 4: Figure S3. — Influence of optCPE in vivo gene transfer on body weight. Body weight of Co7515* PDX bearing mice was measured during tumor growth inhibition. In all animals no systemic toxicities, such as body weight loss, were observed, which strongly indicates the safety of this gene therapeutic approach. (JPG 283 kb) [file 12885_2017_3123_MOESM4_ESM.jpg]
